# Universal test and treat is not associated with sub‐optimal antiretroviral therapy adherence in rural South Africa: the ANRS 12249 TasP trial

**DOI:** 10.1002/jia2.25112

**Published:** 2018-06-11

**Authors:** Collins Iwuji, Nuala McGrath, Alexandra Calmy, Francois Dabis, Deenan Pillay, Marie‐Louise Newell, Kathy Baisley, Kholoud Porter

**Affiliations:** ^1^ Department of Global Health and Infection Brighton and Sussex Medical School Brighton UK; ^2^ Africa Health Research Institute Durban South Africa; ^3^ Institute for Global Health University College London London UK; ^4^ Africa Health Research Institute School of Nursing & Public Health University of KwaZulu‐ Natal KwaZulu‐Natal South Africa; ^5^ Faculty of Medicine and Faculty of Human, Social and Mathematical Sciences University of Southampton Southampton UK; ^6^ Research Department of Epidemiology & Public Health University College London London UK; ^7^ Service des Maladies Infectieuses HIV Unit Hôpitaux Universitaires de Genève Geneva Switzerland; ^8^ Centre INSERM U1219 Bordeaux Population Health Université de Bordeaux Bordeaux France; ^9^ Division of Infection and Immunity University College London London UK; ^10^ Human Development and Health and Global Health Research Institute Faculty of Medicine University of Southampton Southampton UK; ^11^ Department of Infectious Disease Epidemiology Faculty of Epidemiology & Population Health London School of Hygiene and Tropical Medicine London UK

**Keywords:** antiretroviral therapy, HIV, adherence, visual analogue scale, pill count, Africa, test and treat, virologic suppression

## Abstract

**Introduction:**

HIV treatment guidelines now recommend antiretroviral therapy (ART) initiation regardless of CD4 count to maximize benefit both for the individual and society. It is unknown whether the initiation of ART at higher CD4 counts would affect adherence levels. We investigated whether initiating ART at higher CD4 counts was associated with sub‐optimal adherence (<95%) during the first 12 months of ART.

**Methods:**

A prospective cohort study nested within a two‐arm cluster‐randomized trial of universal test and treat was implemented from March 2012 to June 2016 to measure the impact of ART on HIV incidence in rural KwaZulu‐Natal. ART was initiated regardless of CD4 count in the intervention arm and according to national guidelines in the control arm. ART adherence was measured monthly using a visual analogue scale (VAS) and pill counts (PC). HIV viral load was measured at ART initiation, three and six months, and six‐monthly thereafter. We pooled data from participants in both arms and used random‐effects logistic regression models to examine the association between CD4 count at ART initiation and sub‐optimal adherence, and assessed if adherence levels were associated with virological suppression.

**Results:**

Among 900 individuals who initiated ART ≥12 months before study end, median (IQR) CD4 at ART initiation was 350 cells/mm^3^ (234, 503); median age was 34.6 years (IQR 27.4 to 46.4) and 71.7% were female. Adherence was sub‐optimal in 14.7% of visits as measured by VAS and 20.7% by PC. In both the crude analyses and after adjusting for potential confounders, adherence was not significantly associated with CD4 count at ART initiation (adjusted OR for linear trend in sub‐optimal adherence with every 100 cells/mm^3^ increase in CD4 count: 1.00, 95% CI 0.95 to 1.05, for VAS, and 1.03, 95% CI 0.99 to 1.07, for PC). Virological suppression at 12 months was 97%. Optimal adherence by both measures was significantly associated with virological suppression (*p* < 0.001 for VAS;* p* = 0.006 for PC).

**Conclusions:**

We found no evidence that higher CD4 counts at ART initiation were associated with sub‐optimal ART adherence in the first 12 months. Our findings should alleviate concerns about adherence in individuals initiating ART at higher CD4 counts, however long‐term outcomes are needed. ClinicalTrials.gov NCT01509508.

## 1 Introduction

The most recent WHO antiretroviral therapy (ART) guidelines recommend ART initiation regardless of CD4 count 1 based on the findings from two randomized trials of early ART initiation 2, 3. This has now been adopted by South Africa 4, the country with the biggest HIV burden and treatment programme globally. Currently, there is a lack of good quality data on ART adherence at high CD4 counts (CD > 350 cells/mm^3^) in the African setting. In the TEMPRANO trial conducted in Ivory Coast, virological suppression 12 months post‐ART initiation was achieved in 84% and 80% in the immediate (CD4 ≤ 800 cells/mm^3^) and deferred ART (initially CD4 < 200 cells/mm^3^ until 2013, then 500 cells/mm^3^ afterwards) arm, respectively 2. These findings would suggest that adherence levels were equal in both groups, although adherence was not reported in the trial. Findings from two of three studies in the African setting that compared adherence in individuals initiating ART at high CD4 count with those initiating at lower CD4 counts were contradictory 5, 6. Furthermore, these two studies evaluated adherence in patients who were on an ART regimen based mainly on a thymidine analogue backbone (zidovudine or stavudine), known to be less tolerable than tenofovir‐based regimens 7.

ART adherence is critical in order to achieve the third 90 of the UNAIDS 90‐90‐90 target: 90% of all people living with HIV being diagnosed, 90% of diagnosed individuals being on ART, and 90% of those on ART being virologically suppressed 8. However, concern has been expressed that individuals offered ART at higher CD4 counts, with relatively preserved immune function, may not be motivated to adhere to ART as most would be asymptomatic and healthy, hence may not perceive ART to be of immediate benefit to their own health. This could be the case especially in low income settings where people often have competing beliefs about medication taking as well as priorities around economic resources 9.

In this paper, we examine ART adherence in a nested cohort study within the ANRS Treatment as Prevention Trial. The strength of this design is that individuals initiated ART based on the initiation criteria assigned to the cluster in which they were resident rather than self‐selecting when to start ART.

We hypothesized that individuals initiating ART at higher CD4 counts would be more likely to have sub‐optimal adherence than individuals initiating ART at lower CD4 counts. We quantified adherence using two different adherence measurement tools. We examined whether CD4 count at ART initiation was associated with sub‐optimal adherence during the first 12 months of ART and assessed which measures of adherence adequately predicted virological suppression at 12 months.

## 2 Methods

### 2.1 Ethics statement

The main trial was approved by the Biomedical Research Ethics Committee (BFC 104/11) of the University of KwaZulu‐Natal and the Medicines Control Council of South Africa. (ClinicalTrials.gov: NCT01509508; South African National Clinical Trials Register: DOH‐27‐0512‐3974). The nested cohort study received additional approval from University College London Research Ethics Committee (Project ID: 6604/001). All participants provided written or witnessed thumb‐print informed consent.

### 2.2 Study design and participants

The investigations were conducted within a prospective cohort study nested within a cluster‐randomized trial implemented in 22 clusters (2 × 11) from March 2012 to June 2016 to investigate the impact of ART on population HIV incidence in the Hlabisa sub‐district in rural KwaZulu‐Natal 10. This is a rural setting with scattered homesteads and an estimated HIV prevalence of 30.5% 11. Control arm participants were offered ART according to the South African guidelines (CD4 count ≤350 at trial start, then CD4 count ≤500 from January 2015). Those in the intervention arm were offered ART regardless of CD4 count. The trial protocol has been described previously 12. In this cohort study, sub‐optimal adherence was examined according to CD4 count at ART initiation, irrespective of arm in trial. Individuals were eligible for inclusion in the cohort if aged ≥16 years, and had initiated ART at least 12 months prior to database closure on 30 June 2016.

### 2.3 Procedures

Six‐monthly home‐based HIV counselling and testing (HCT) using rapid test technology was offered to resident members of the trial communities using a serial testing algorithm 13. Individuals identified HIV positive were referred to trial clinics located in each of the 22 clusters. HIV‐positive participants enrolled in trial clinics were asked to provide written consent to complete case report forms and provide blood specimens for viral load (VL) testing. ART was offered according to cluster allocation. All participants had point‐of‐care CD4 measurement (Alere Pima CD4 test, Alere, Waltham, MA, US); those eligible for ART attended adherence and ART literacy sessions and were offered ART within two weeks of the baseline visit, or sooner if severely immunocompromised. The single tablet regimen, Atripla (comprising tenofovir, emtricitabine & efavirenz) was used for first‐line ART, except if clinically contraindicated such as in renal disease. Second‐line ART was informed by the results of genotypic resistance tests in participants failing first‐line ART (VL > 1000 copies/ml measured three months apart after ≥six months on ART).

Participants receiving ART were evaluated monthly for adherence measurement and ART prescription. Scheduled safety monitoring of blood (urea, electrolytes, creatinine, liver function tests, full blood count) and HIV VL measurements (Abbott m2000 RealTime System, Abbott Molecular, Des Plaines, IL, US) occurred at the first visit, three and six months after ART initiation, and every 6 months thereafter. Participants were also encouraged to attend the clinic at unscheduled visits if they had clinical complaints. Patients not yet eligible for ART in the control clusters were asked to return to the study clinic in four to six months for reassessment of ART eligibility. A participant missing a clinic appointment was contacted by telephone, and, when possible, a new appointment was scheduled. Those not contacted by phone were followed up with home visits carried out by trackers. Participants who did not attend within 90 days of their last clinic appointment and who could not be contacted were considered lost to follow‐up.

### 2.4 Definition of outcome and exposure variables

Adherence was measured using both a visual analogue scale (VAS) and pill counts (PC) at each scheduled visit.

The VAS was represented by a horizontal line with ends at 0 and 100. Participants were asked to put a mark on the scale which best reflected their adherence in the previous four days. Adherence was categorized as sub‐optimal if the VAS was <95%.

PC adherence was calculated [(N tablets issued − N tablets returned)/N tablets expected to have been taken]*100. Adherence was considered sub‐optimal if PC adherence was <95% or >105%.

CD4 cell count at ART initiation was the primary exposure variable.

### 2.5 Statistical analysis

Baseline characteristics were tabulated by sex.

Adherence at each visit was plotted over the first 12 months after ART initiation; during this period, adherence was expected to be documented at 14 visits (2, 4, 8, 12, 16, 20, 24, 28, 32, 36, 40, 44, 48, 52 weeks post‐ART initiation) for those who remained in the trial for the 12 months. The number of expected visits was lower among those who exited the trial earlier than 12 months.

Random effects logistic regression was used to examine the association between CD4 count at initiation and sub‐optimal adherence at each visit. All models included a priori an indicator for trial arm, a fixed effect for time since ART start, a random coefficient (slope) for time at the individual level, and random intercepts at both the clinic and the individual‐within‐clinic levels.

CD4 count at ART initiation was analysed as a continuous covariate. In order to allow for non‐linear relationships between CD4 count and adherence, we used fractional polynomial (FP) functions 14. Fractional polynomials provide a flexible way to model the shape of the relationship of a continuous variable with the outcome. We used a set of defined powers (−2, −1, −0.5, 0.5, 1, 2 and ln(x)) and a maximum of two power terms in the model. The differences in model deviances were compared; the linear model was used if the improvement in fit was not statistically significant at *p* < 0.05. Time in trial and age at ART initiation were handled in a similar manner. Other continuous exposure variables (distance to clinic, self‐reported health status) were categorized, a priori, into binary variables above and below their median values. We used the validated Patient Health Questionnaire (PHQ4) scale published in the literature for screening of depression 15.

In the final multivariable analysis, we adjusted for potential confounders commonly cited in the literature 16, 17. We tested for interactions between CD4 count and trial arm, CD4 count and time in trial, and CD4 count and sex, to assess whether the effect of CD4 count on adherence depended on trial arm, time or on sex. Likelihood ratio tests were used to derive *p*‐values.

We also assessed whether mean VAS or PC score in each individual during the first 12 months of ART was associated with virological suppression at 12 months. Participants were considered to be virologically suppressed if their viral load was below 400 copies/ml; the viral load measurement taken closest to the 12‐month time point, within a ±three‐month window, was used for the assessment. Mean adherence scores were calculated for each participant by taking the mean of the observed adherence scores at each visit. Adherence measures were classified into three (VAS) and four (PC) categories to explore relationship with virological suppression. As a sensitivity analysis, we examined the association of mean adherence during the first six months on ART with virological suppression at six months.

All statistical analyses were undertaken using Stata 15 (StataCorp LLC, College Station, Texas 77845, USA).

## 3 Results

### 3.1 Cohort characteristics

**Figure 1:**
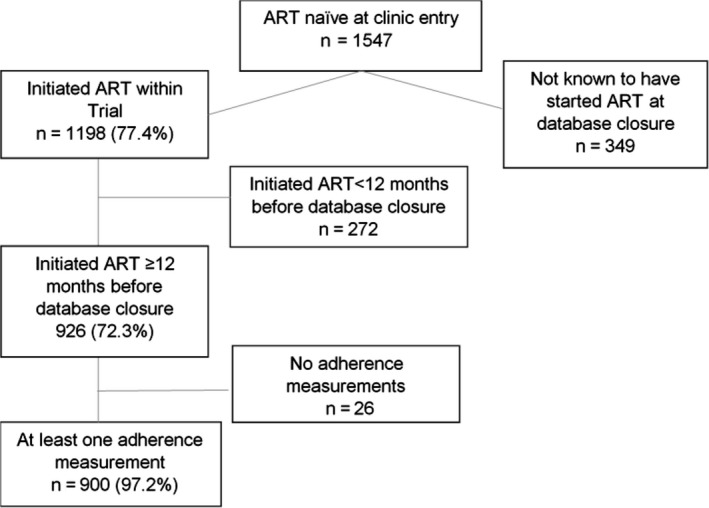
Flow chart of cohort.

A total of 1547 ART‐naïve (self‐reported never being on ART) individuals were enrolled in trial clinics, of whom 1198 initiated ART. Of the 926 who initiated ART at least 12 months before database closure, 900 had at least one adherence measurement (VAS or Pill count) during the 12‐month period and were included in the analyses (Figure 1).

Of the 900 individuals who were included in the analysis, 72% were female. Median age was 34.6 (IQR 27.4 to 49.5); females were younger than males (median 33.3 years *vs*. 36.7 years, respectively). Educational attainment was low, with 42% of women and 45% of men having only primary education. A large proportion of the population was unemployed (84% women *vs*. 73% men). The median CD4 count at ART initiation was 350 (IQR 234 to 503) (Table 1).

**Table 1:** Characteristics of individuals included in the analysis during the first 12 months’ adherence analysis using visual analogue scale and pill count

	Female N = 645 (71.7%) n (% of N)	Male N = 255 (28.3%) n (% of N)	Total N = 900
Clinical characteristics			
CD4 at initiation Median (IQR)	374 (254, 525)	311 (205, 451)	350 (234, 503)
≤350	295 (45.7)	154 (60.4)	449 (49.9)
350 to 500	166 (25.7)	56 (22.0)	222 (24.7)
>500	181 (28.1)	45 (17.7)	226 (25.1)
Missing	3 (0.5)	0 (0.0)	3 (0.3)
Viral Load at first clinic visit (Log_10_ copies/ml)			
Median (IQR)	4.4 (3.8, 5.1)	4.8 (4.1, 5.4)	4.5 (3.8, 5.2)
Age at initiation (years)			
Median age (IQR)	33.3 (26.0, 45.0)	36.7 (29.9, 49.5)	34.6 (27.4,46.4)
16 to 29	254 (39.4)	66 (25.9)	320 (35.6)
30 to 39	170 (26.6)	82 (32.2)	252 (28.0)
40 to 49	108 (16.7)	45 (17.7)	153 (17.0)
>50	112 (17.4)	62 (24.3)	174 (19.3)
Missing	1 (0.2)	0 (0.0)	1 (0.1)
Educational attainment			
Primary or less	274 (42.5)	116 (45.5)	390 (43.3)
Some secondary	344 (53.3)	127 (49.8)	471 (52.3)
Completed secondary or higher	23 (3.6)	12 (4.7)	35 (3.9)
Missing	4 (0.6)	0 (0.0)	4 (0.4)
Marital status			
Never married	564 (87.4)	215 (84.3)	779 (86.6)
Married	45 (7.0)	33 (12.9)	78 (8.7)
Divorced/separated	33 (5.1)	7 (2.8)	40 (4.4)
Missing	3 (0.5)	0 (0.0)	3 (0.3)
Employment status			
Employed	75 (11.6)	62 (24.3)	137 (15.2)
Student	29 (4.5)	6 (2.4)	35 (3.9)
Unemployed	540 (83.7)	186 (72.9)	726 (80.7)
Missing	1 (0.2)	1 (0.4)	2 (0.2)
Trial arm			
Intervention	278 (43.1)	110 (43.1)	388 (43.1)
Control	367 (56.9)	145 (56.9)	512 (56.9)
Food insecurity			
Yes	420 (65.1)	149 (58.4)	569 (63.2)
No	210 (32.6)	101 (39.6)	311 (34.6)
Do not know	6 (0.9)	4 (1.6)	10 (1.1)
Missing	9 (1.4)	1 (0.4)	10 (1.1)

IQR, interquartile range.

### 3.2 Comparison of adherence measurements

Of the 7945 visits where participants had both VAS and PC measurements, the two measurements were concordant in 6493 (81.7%) of visits, with adherence classified as optimum according to both measures in 73.5% of visits, and suboptimal in 8.2% of visits. VAS and PC were discordant in 18.3% of visits; adherence was optimal on PC but sub‐optimal on VAS in 5.8% of visits, and sub‐optimal on PC but optimal on VAS in 12.5% of visits.

### 3.3 Association between CD4 count at initiation and visual analogue scale adherence <95% during the first 12 months

The 900 participants had 8874 (77.1%) visits with VAS adherence measurements, of the 11,507 expected visits in the 12‐month period. VAS adherence was optimal (≥95%) in 7566 (85.3%) of these 8874 visits (Figure S1). The median number of visits per individual was 11 (IQR 10 to 12).

In the crude analysis, and after adjusting for potential confounders, there was no evidence of an association between CD4 count at ART initiation and sub‐optimal VAS adherence during the first 12 months on ART (adjusted (a)OR for linear trend in sub‐optimal adherence with every 100 cells/mm^3^ increase in CD4 count = 1.00, 95% CI 0.95 to 1.05, *p* = 0.96; Table 2). The results of the FP models showed that the linear model adequately described the relationship between CD4 count and VAS adherence. There was no evidence that the effect of CD4 count on VAS adherence differed between trial arms, between men and women, or with time in the trial (*p*‐values for interaction = 0.06, 0.17, and 0.29, respectively).

**Table 2:** Association between CD4 count at initiation and other factors with <95% visual analogue scale adherence during the first 12 months of ART

Characteristics	Adherence <95% N visits/total visits	¥Crude odds ratio (95% CI)	*p* value	^&^Adjusted odds ratio (95% CI)	*p* value
CD4 at Initiation (cells/mm^3^) n = 8866					
≤350	679/4432 (15.3)				
350 to 500	318/2231 (14.3)	0.97 (0.93 to 1.02)^a^	0.204	1.00 (0.95 to 1.05)^a^	0.963
>500	308/2203 (14.0)				
Age at initiation n = 8864					
16 to 29	476/2847 (16.7)				
30 to 39	355/2520 (14.1)	1.01 (0.97 to 1.05)^b^	0.625	0.98 (0.93 to 1.04)^b^	0.464
40 to 49	222/1654 (13.4)				
>50	255/1843 (13.8)				
Sex n = 8874			<0.0001		<0.0001
Female	811/6500 (12.5)	1		1	
Male	497/2374 (20.9)	2.21 (1.76 to 2.77)		2.29 (1.80 to 2.90)	
Education n = 8830					0.983
Primary or less	563/4045 (13.9)	1		1	
Some secondary	693/4441 (15.6)	1.01 (0.81 to 1.26)		1.00 (0.76 to 1.30)	
At least completed secondary	46/344 (13.4)	0.92 (0.51 to 1.65)		0.94 (0.51 to 1.75)	
Marital status n = 8841			0.417		0.303
Never been married	1150/7616 (15.1)	1		1	
Married	106/818 (13.0)	0.90 (0.60 to 1.33)		0.73 (0.48 to 1.12)	
Divorced/separated	46/407 (11.3)	0.71 (0.41 to 1.22)		0.79 (0.45 to 1.39)	
Employment status n = 8852			0.743		0.810
Employed	217/1396 (15.5)	1		1	
Student	54/309 (17.5)	1.02 (0.55 to 1.89)		1.23 (0.65 to 2.33)	
Unemployed	1033/7147 (14.5)	0.90 (0.67 to 1.21)		1.03 (0.77 to 1.39)	
First line Regimen n = 8835			<0.0001		0.0005
Separate tablet regimen	91/382 (23.8)	1		1	
Single tablet regimen	1204/8453 (14.2)	0.72 (0.61 to 0.85)		0.40 (0.24 to 0.67)	
ART treatment perception					
Agree that ART will improve health n = 8760			0.854		0.641
Yes	1227/8395 (14.6)	1		1	
No	22/128 (17.2)	1.20 (0.52 to 2.79)		1.22 (0.49 to 3.01)	
Do not know	40/237 (16.9)	1.14 (0.59 to 2.21)		1.41 (0.65 to 3.04)	
Worried about side effects of ART n = 8703			0.599		0.859
Yes	1075/7409 (14.5)	1		1	
No	65/433 (15.0)	1.08 (0.65 to 1.80)		0.96 (0.54 to 1.69)	
Do not know	138/861 (16.0)	0.84 (0.57 to 1.22)		0.89 (0.60 to 1.33)	
Agree that ART will reduce transmission n = 8626			0.193	–	–
Yes	889/6627 (13.4)	1			
No	120/705 (17.0)	1.37 (0.91 to 2.06)			
Do not know	245/1294 (18.9)	1.26 (0.90 to 1.77)			
HIV status disclosure to anyone n = 8739			0.891		0.368
Yes	1108/7485 (14.8)	1		1	
No	189/1254 (15.1)	0.98 (0.72 to 1.34)		0.86 (0.63 to 1.19)	
HIV status disclosure to current partner n = 8574			0.05	–	–
Yes	724/4739 (15.3)	1			
No partner disclosure	351/2487 (14.1)	0.84 (0.66 to 1.08)			
No partner	195/1348 (14.5)	0.91 (0.67 to 1.24)			
** **Food insecurity n = 8783			0.639		0.057
Yes	912/5668 (16.1)	1		1	
No	377/3025 (12.5)	0.89 (0.71 to 1.14)		0.76 (0.60 to 0.97)	
Do not know	10/90 (11.1)	0.84 (0.27 to 2.61)		2.95 (0.21 to 41.94)	
Psychological distress (PHQ4) n = 8597			0.547		–
None	997/7023 (14.2)	1		–	
Mild	244/1337 (17.7)	1.11 (0.79 to 1.56)			
Moderate	18/103 (17.5)	1.34 (0.52 to 3.39)			
Severe	21/94 (22.3)	1.86 (0.70 to 4.95)			
Self to reported health status n = 8863			0.535		0.975
≤80	801/5474 (14.6)	1		1	
>80	506/3389 (14.9)	0.93 (0.74 to 1.17)		1.00 (0.79 to 1.27)	
Distance from home to trial clinic (km) n = 8874			0.804		0.607
≤1.3	683/4433 (15.4)	1		1	
>1.3	625/4441 (14.1)	1.03 (0.82 to 1.29)		0.94 (0.75 to 1.18)	
Time in study (months) n = 8874					
≤6	711/4927 (14.4)	1.01 (0.99 to 1.03)	0.284	1.01 (0.98 to 1.03)^c^	0.536
>6	597/3947 (15.1)				
Trial arm n = 8874			0.452		0.506
Control	617/3852 (16.0)	1		1	
Intervention	691/5022 (13.8)	0.79 (0.43 to 1.45)		0.82 (0.45 to 1.49)	

¥ORs estimated from random effects logistic regression, with a fixed effect for time, a random coefficient for time at the individual level, and random intercepts at both the cluster and the individual‐within‐cluster level. &adjusted for age, sex, marital status, employment, whether on fixed dose combination of ART, food insecurity, distance to clinic, worried about side‐effects, agree that ART will improve health, status disclosure to anyone and self‐reported health status and trial arm. ^a^Odds ratio for linear trend in sub‐optimal adherence with every 100‐unit increase in CD4 count at initiation. ^b^Odds ratio for linear trend in sub‐optimal adherence with every 5‐year increase in age. ^c^Odds ratio for linear trend in sub‐optimal adherence with every month on ART. Distance to the nearest TasP clinic: obtained by measuring the distance as the crow flies from the participant's home (GPS coordinates) to the trial clinic (GPS coordinates) in their cluster. Depression (assessed using the Patient Health Questionnaire (PHQ)‐4 scale rated as normal (0 to 2), mild (3 to 5), moderate (6 to 8) and severe (9 to 12), 15. Self‐reported health status (as measured using a scale ranging from 0 to 100% in which 0 represents poor health and 100% represents excellent health). Food insecurity (as measured by whether skipped meals in last 12 months or not). ART treatment perception (through three questions concerning the participant's attitudes about ART).

In the final model, there was strong evidence of an association of male sex with sub‐optimal VAS adherence (aOR 2.29, 95% CI 1.80 to 2.90, *p* < 0.001). Being on a single tablet ART regimen was associated with a lower odds of sub‐optimal adherence (aOR 0.40, 95% CI 0.24 to 0.67, compared with those on separate tablet regimen; *p* < 0.001). In addition, there was some evidence that individuals who did not have food insecurity were less likely to have sub‐optimal adherence (aOR 0.76, 95% CI 0.60 to 0.97, *p* = 0.06). There was no evidence of association of time on ART (*p* = 0.54), or of trial arm (*p* = 0.51), with sub‐optimal adherence as measured by VAS.

### 3.4 Association between CD4 count at initiation and sub‐optimal pill count adherence during the first 12 months

Of the 900 participants in the current study, four had no pill count adherence measurements. The 896 participants had PC adherence measurements at 8014 (69.8%) of the 11,475 expected visits in the 12‐month period. PC adherence was optimal in 6352 (79.3%) of these visits, and was >105% in 5.9% of visits (Figure S2). The median number of visits with PC adherence data per individual was 11 (IQR 9 to 12).

In the crude analysis, and after adjusting for potential confounders, there was no evidence of an association between CD4 count at ART initiation and sub‐optimal adherence as measured by PC during the first 12 months on ART (aOR for linear trend in sub‐optimal adherence with every 100 cells/mm^3^ increase in CD4 count = 1.03, 95% CI 0.99 to 1.07, *p* = 0.21). The results of the FP models showed that the linear model adequately described the relationship between CD4 count and PC adherence. There was no evidence that the effect of CD4 count on PC adherence differed between trial arms, between men and women, or with time in the trial (*p*‐values for interaction = 0.26, 0.09, and 0.22, respectively).

In the final model, as with VAS adherence, there was strong evidence of an association of male sex with sub‐optimal PC adherence. Similarly, being on a single tablet ART regimen was associated with a lower odds of sub‐optimal adherence. Unlike with VAS adherence, there was strong evidence that sub‐optimal PC adherence increased with increasing time on ART (aOR for linear trend in sub‐optimal adherence with every month on ART = 1.04, 95% CI 1.02 to 1.06, *p* < 0.001). However, there was no evidence of an association with trial arm (*p* = 0.17) (Table 3).

**Table 3:** Association between CD4 count at initiation and sub‐optimal pill count adherence during the first 12 months of ART

Characteristics	Adherence <95%/>105 N visits/total visits	¥Crude odds ratio (95% CI)	*p* value	^&^Adjusted odds ratio (95% CI)	*p* value
CD4 at initiation (cells/mm^3^) n = 8006					
350	851/4016 (21.2)				
350 to 500	400/2009 (19.9)	1.00 (0.96 to 1.03)^a^	0.830	1.03 (0.99 to 1.07)^a^	0.205
>500	407/1981 (20.6)				
Age at initiation n = 8005					
16 to 29	580/2559 (22.7)				
30 to 39	464/2313 (20.1)	0.99 (0.96 to 1.03)^b^	0.632	0.96 (0.91 to 1.01)^b^	0.085
40 to 49	286/1482 (19.3)				
>50	330/1651 (20.0)				
Sex n = 8014			<0.0001		<0.0001
Female	1057/5962 (17.7)	1		1	
Male	605/2052 (29.5)	2.23 (1.83 to 2.71)		2.41 (1.95 to 2.97)	
Educational attainment n = 7972			0.469		0.218
Primary or less	732/3682 (19.9)	1		1	
Some secondary	868/3974 (21.8)	1.06 (0.87 to 1.29)		0.98 (0.77 to 1.24)	
At least completed secondary	51/316 (16.1)	0.78 (0.47 to 1.31)		0.62 (0.35 to 1.08)	
Marital status n = 7983			0.886		0.543
Never been married	1435/6872 (20.9)	1		1	
Married	142/753 (18.9)	0.93 (0.67 to 1.30)		0.87 (0.61 to 1.25)	
Divorced/separated	74/358 (20.7)	0.93 (0.59 to 1.48)		1.17 (0.73 to 1.88)	
Employment status n = 7992			0.391		0.956
Employed	297/1258 (23.6)	1		1	
Student	56/274 (20.4)	0.97 (0.56 to 1.69)		1.01 (0.57 to 1.80)	
Unemployed	1300/6460 (20.1)	0.84 (0.65 to 1.09)		0.97 (0.75 to 1.25)	
First line regimen n = 7977			0.013		0.019
Separate tablet regimen	74/285 (26.0)	1		1	
Single tablet regimen	1581/7692 (20.6)	0.82 (0.70 to 0.96)		0.56 (0.34 to 0.90)	
ART treatment perception					
Agree that ART will improve health n = 7908			0.589		0.499
Yes	1567/7569 (20.7)	1		1	
No	29/111 (26.1)	1.44 (0.68 to 3.03)		1.61 (0.73 to 3.54)	
Do not know	51/228 (22.4)	1.13 (0.64 to 1.98)		1.09 (0.56 to 2.15)	
Worried about side effects of ART n = 7858			0.779		0.202
Yes	1383/6644 (20.8)	1		1	
No	74/400 (18.5)	0.94 (0.60 to 1.48)		0.69 (0.42 to 1.13)	
Do not know	178/814 (21.9)	1.11 (0.80 to 1.54)		1.15 (0.81 to 1.63)	
Agree that ART will reduce transmission n = 7781			0.778	–	–
Yes	1225/5922 (20.7)	1			
No	132/646 (20.4)	1.13 (0.78 to 1.62)			
Do not know	256/1213 (21.1)	1.07 (0.80 to 1.43)			
HIV status disclosure to anyone n = 7888			0.247		0.603
Yes	1386/6752 (20.5)	1		1	
No	259/1136 (22.8)	1.17 (0.90 to 1.54)		1.08 (0.82 to 1.42)	
HIV status disclosure to current partner n = 7743					
Yes	893/4277 (20.9)	1		–	
No	450/2240 (20.1)	0.91 (0.73 to 1.13)			
Not applicable (No partner)	272/1226 (22.2)	1.10 (0.84 to 1.45)			
Food Insecurity n = 7035			0.860		0.440
Yes	1076/5090 (21.1)	1		1	
No	561/2764 (20.3)	1.02 (0.83 to 1.25)		0.87 (0.71 to 1.07)	
Do not know	14/81 (17.3)	0.78 (0.30 to 2.01)		0.99 (0.09 to 11.43)	
Psychological distress (PHQ4) n = 7758			0.108		
None	1301/6432 (20.2)	1			
Mild	281/1147 (24.5)	0.91 (0.67 to 1.23)		–	–
Moderate	19/94 (20.2)	1.07 (0.46 to 2.47)			
Severe	27/85 (31.8)	2.84 (1.20 to 6.74)			
Self to reported health status n = 8003			0.916		0.843
≤80	1004/4917 (20.4)	1		1	
>80	653/3086 (21.2)	1.01 (0.83 to 1.23)		1.02 (0.83 to 1.26)	
Distance from home to trial clinic (Km) n = 8014			0.634		0.396
≤1.3	864/3923 (22.0)	1		1	
>1.3	798/4091 (19.5)	0.95 (0.78 to 1.16)		0.92 (0.75 to 1.12)	
Time (months) n = 8007					
≤6	854/4494 (19.0)	1.04 (1.02 to 1.06)	<0.001	1.04 (1.02 to 1.06)^c^	<0.001
>6	805/3513 (22.9)				
Trial arm n = 8014			0.246		0.173
Control	779/3384 (23.0)	1		1	
Intervention	883/4630 (19.1)	0.77 (0.49 to 1.20)		0.74 (0.48 to 1.13)	

¥ORs estimated from random effects logistic regression, with a fixed effect for time, a random coefficient for time at the individual level, and random intercepts at both the cluster and the individual‐within‐cluster level. &adjusted for age, sex, marital status, employment, whether on fixed dose combination of ART, food insecurity, distance to clinic, worried about side‐effects, agree that ART will improve health, status disclosure to anyone and self‐reported health status and trial arm. *Odds ratio for linear trend in sub‐optimal adherence with every 100‐unit increase in CD4 count at initiation. #Odds ratio for linear trend in sub‐optimal adherence with every 5‐year increase in age. βOdds ratio for linear trend in sub‐optimal adherence with every month on ART. Distance to the nearest TasP clinic: obtained by measuring the distance as the crow flies from the participant's home (GPS coordinates) to the trial clinic (GPS coordinates) in their cluster. Depression (assessed using the Patient Health Questionnaire (PHQ)‐4 scale rated as normal (0 to 2), mild (3 to 5), moderate (6 to 8) and severe (9 to 12), 15. Self‐reported health status (as measured using a scale ranging from 0 to 100% in which 0 represents poor health and 100% represents excellent health). Food insecurity (as measured by whether skipped meals in last 12 months or not). ART treatment perception (through three questions concerning the participant's attitudes about ART).

### 3.5 Relationship between adherence and virological suppression at 12 months

**Figure 2:**
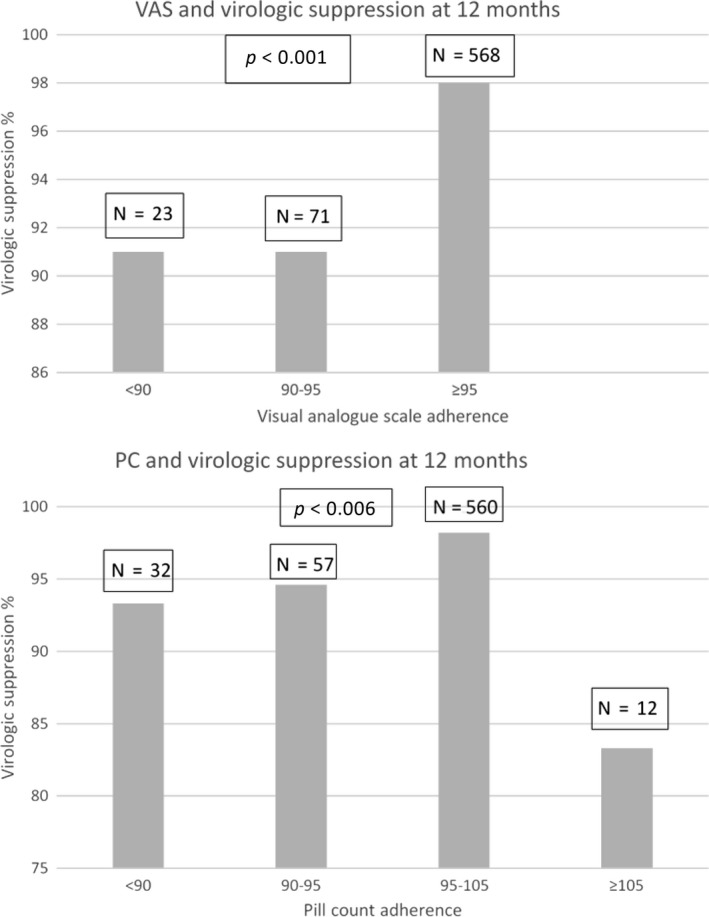
Relationship between mean adherence levels over 12 months measured by visual analogue scale (upper panel) and pill count (lower panel) and virological suppression at 12 months.

Of 664 individuals with viral load data at 12 months, 644 (97%) achieved virological suppression. Of the 568 individuals with mean VAS adherence ≥95%, 557 (98%) achieved virological suppression at 12 months compared to 86/94 (91%) in those with <95% adherence (*p* < 0.001; Figure 2). When adherence was measured by PC, optimal adherence (95 to 105%) was also predictive of higher odds of virological suppression (98%) compared to those with lower levels of adherence (Figure 2). Of note, only 83% with adherence ≥105% as measured by PC achieved virological suppression at 12 months. Similar patterns were seen with virological suppression at six months (Figure S3).

## 4 Discussion

In this cohort analysis of participants enrolled in a cluster randomized trial, the majority of whom were female, we found no evidence of a significant association between CD4 count at ART initiation and sub‐optimal adherence measured by either VAS or PC during the first 12 months of ART. Adherence measured by VAS and PC was sub‐optimal in 15% and 21% of visits respectively during the first 12 months of ART. Virological suppression was high overall with optimal adherence by both measures being associated with virological suppression at 12 months.

We identified only two studies in the African setting, the first a retrospective and the other a cross‐sectional study that 5, 6 assessed risk factors for adherence in individuals who initiated ART at CD4 count >350 cells/mm^3^ compared to those with lower CD4 counts. The retrospective study 5 reported an association between higher CD4 count at initiation and adherence <95% whilst the cross‐sectional study 6 found no association between CD4 count at initiation and adherence. In both studies, the reference group comprised individuals with advanced HIV disease based on the reported median CD4 count at ART initiation. Our cohort comprised individuals with a higher median CD4 count at ART initiation than in those studies and findings corroborate that seen in high income countries reported in the systematic review by Bock et al. 18. WHO recommends universal test and treat for HIV 1; South Africa has already adopted this recommendation 4 but there are no data on adherence in people initiating ART at high CD4 counts (CD4 > 350) in the African setting. With the new treatment guidelines, the median CD4 count at which individuals initiate ART is likely to rise to levels observed in our cohort. However, a meta‐analysis covering the period from January 2002 to Dec 2013 showed that the CD4 count at presentation for HIV care has increased in South Africa but the CD4 count at ART initiation has remained unchanged at a mean of 123 cells/mm^3^ 18.

One of the WHO's early warning indicators for development of HIV drug resistance is the proportion of pills picked up on time during the first 12 months of ART which serves as a proxy for adherence. The proportion of study visits with optimal adherence during the first 12 months of ART falls just under the >90% WHO recommendation 19 despite the high proportion of participants who were virologically suppressed.

Using either adherence measure, men had more than double the odds of sub‐optimal adherence compared with women, similar to findings reported in two studies in Tanzania 20 and South Africa 21. We observed a high out‐migration rate which was cyclical in nature within the TasP trial. In the population adjacent to the TasP communities, a higher outmigration rate has been reported for men compared to women 22. This could have contributed to the poorer adherence seen in men than women in our study. The majority of studies have reported no sex difference with respect to adherence 23, 24, 25, 26, 27, 28, with one meta‐analysis reporting a marginal association of male sex with higher adherence 17.

Individuals who were on a single tablet ART regimen (fixed dose combination of tenofovir, emtricitabine and efavirenz) compared to those taking separate tablet regimen (mainly zidovudine, lamivudine and efavirenz) had a lower odds of sub‐optimal adherence. This could be due to the better tolerability profile of tenofovir‐based ART regimen than zidovudine‐based ART combination 7 Furthermore, the once daily tenofovir based ART combination could have made adherence easier than zidovudine‐based ART which had to be taken twice daily.

We found that food insecurity was associated with sub‐optimal adherence, similar to findings in Namibia amongst individuals attending a public ART programme 29. The relationship between food insecurity and poor adherence has also been reported in high‐income countries 30, 31. Patients who have missed doses have often cited not having food at home as a reason for missing doses because of the prevailing perception that it is bad to take their drugs on an empty stomach. This anecdotal observation has been confirmed in formal qualitative studies 32, 33 and should be discussed when preparing patients for ART initiation.

Although there is no gold standard measure of adherence 34, we found both VAS and Pill count adherence to be predictive of virological suppression. However, there were differences between both tools. Although we found high agreement between the two measures, overall adherence as measured by PC was lower than that of VAS suggesting there is an intrinsic error associated with the use of each tool 35. PC adherence was missing in 30% of visits whilst 23% of visits had missing VAS adherence. Participants frequently forgot to bring in their pill bottles, or the health care provider did not take the measure. Pill count adherence was >105% in 6% of visits; this apparent “over‐adherence” predicted poor virological suppression so may likely have been owing to participants discarding pills prior to their clinic appointment 35. The ease of use of the VAS would suggest it is preferable in the busy clinical setting of HIV clinics in South Africa and elsewhere. However, unlike with VAS adherence, we found an association between increased time on ART and increased odds of suboptimal adherence when using PC adherence in the relatively short duration of our study. A recent multicentre prospective study showed that good adherence during the first four months of ART made undetectable viral load more than three times likely over a 12 year period 36. This highlights that adherence support needs to start as soon as individuals initiate ART and continue lifelong.

This research study has a few limitations. We included all individuals who would have been on ART for 12 months by the time of database closure, rather than restricting our analyses to only those individuals who remained in the trial for the 12‐month period. This reduces the likelihood of selection bias. The downside, however, was the large numbers of missing visits observed as individuals only contributed data for the duration they were present in the study. If disengagement from care was related to poor adherence, then we could have overestimated adherence and virological suppression in the trial. We examined adherence during the first 12 months of ART, hence our findings cannot be extrapolated to adherence lifelong.

The main strength of our analysis is that it was nested within a cluster‐randomized trial, so that individuals initiated ART based on the initiation criteria assigned to the cluster in which they were resident, rather than self‐selecting when to start ART. This could have mitigated against any bias that might be introduced if individuals choosing to start ART at higher CD4 counts were more motivated and hence more likely to adhere. To the best of our knowledge, this is the first study examining the association between CD4 count at ART initiation and sub‐optimal adherence in individuals initiating ART at higher CD4 counts in the African Setting.

## 5 Conclusions

We found no evidence of a significant relationship between CD4 count at ART initiation and sub‐optimal adherence during the first 12 months of ART, using two different measurements of adherence. With two large trials showing individual health benefits of initiating ART early 2, 3 and the WHO 2015 ART guidelines recommending HIV treatment regardless of CD4 count 1, a policy already adopted by South Africa 4, this result should alleviate any concern about adherence in individuals initiating ART at higher CD4 counts, at least during the first 12 months after ART initiation. This study also provides much needed evidence on the relationship between adherence and virologic suppression in this setting and supports the UNAIDS 90‐90‐90 target.

## Competing interests

CI received honoraria for consulting services rendered to Gilead Sciences. All other authors declare that they have no conflicts of interest.

## Author contributions

CI designed and implemented the study. CI did the statistical analyses with support from KB and KP. CI wrote the initial draft of the manuscript. CI, KB, NM, AC, FD, DP, MLN and KP contributed to the interpretation and presentation of the findings. All authors approved the final version of the manuscript for submission.

## Supporting information


**Figure S1.** VAS adherence at each visit, among patients with CD4 count <350 at ART initiation (top‐1A) and CD4 count ≥350 (bottom‐1B).
**Figure S2.** Pill count adherence at each visit, among patients with CD4 count <350 at ART initiation (top‐2A) and CD4 count ≥350 (bottom‐2B).
**Figure S3.** Viral suppression and mean adherence over 6 months as measured by VAS (2A) and pill counts (2B).
