# Supplementary material for: Universal test and treat is not associated with sub‐optimal antiretroviral therapy adherence in rural South Africa: the ANRS 12249 TasP trial
Source: J Int AIDS Soc. 2018 Jun 11;21(6):e25112. doi: 10.1002/jia2.25112 (PMC5995313; doi:10.1002/jia2.25112)
Supplement: Supplementary file 1 — Figure S1. VAS adherence at each visit, among patients with CD4 count <350 at ART initiation (top‐1A) and CD4 count ≥350 (bottom‐1B). Figure S2. Pill count adherence at each visit, among patients with CD4 count <350 at ART initiation (top‐2A) and CD4 count ≥350 (bottom‐2B). Figure S3. Viral suppression and mean adherence over 6 months as measured by VAS (2A) and pill counts (2B). [file JIA2-21-e25112-s001.docx]

**Supplementary Figure 1: VAS adherence at each visit, among patients with CD4 count <350 at ART initiation (top-1A) and CD4 count ≥350 (bottom-1B)**

**Supplementary Figure 2: Pill count adherence at each visit, among patients with CD4 count <350 at ART initiation (top-2A) and CD4 count ≥350 (bottom-2B)**

**Supplementary Figure 3: Viral suppression and mean adherence over 6 months as measured by VAS (2A) and pill counts (2B)**

P<0.001

665

76
